# Report of Dr. Caleb Green, of Homer, N. Y., to the Medical Association of Southern Central New York, on the Qualifications Requisite for Commencing the Study of Medicine

**Published:** 1850-03

**Authors:** 


*Report of Dr. Caleb Green, of Homer, N. Y., to the Medical Association of Southern Central New York, on the qualifications requisite for commencing the study of medicine.*

We copy from the Transactions of the Association of Southern Central New York, the following sensible remarks on the important subject of the preliminary education of Medical Students.—Ed. Buffalo Med. Jour.

In the investigation of the subject of qualifications requisite for commencing the study of medicine, the Committee have taken into consideration: 1st, the nature and extent of the literary and scientific acquirements which should be demanded of the Student; and 2dly, the moral character which he is expected to possess.

In examining the first department of this inquiry, the Committee are led, directly, to consider the *object of education.* This they regard to be twofold: viz., 1st, the discipline of the mind,—the formation of certain habits of study, observation and reflection; and 2dly, the acquisition of a certain amount of practical information.

The first object may be gained by the pursuit of almost any department of knowledge, provided the student has the advantage of judicious school discipline. But considering the fact that a knowledge of all similar branches of learning is not equally demanded by all professions, we must inquire what are those subjects, concerning which the medical student should have specific and positive information, and whether the acquisition of this knowledge will secure the requisite discipline.

One of the first and most important desiderata to be secured in the educational course, in connection with the specific information acquired, is a certain clearness and definiteness of apprehension. "The student," as a certain writer observes, "ought not merely to *learn*, but to *know*, and *to know that he knows*." We are all probably aware, that in medicine, owing to a want of mathematical certainty in some departments of the science, there is a constant tendency to vague generalization, and to the formation of opinions which will not endure the test of rigid scrutiny. Now this tendency should be combatted by such a course of discipline as would render the student dissatisfied with any thing short of clear conceptions of the bearings of the subject under consideration. We conceive that this mental qualification will be most effectually secured by a thorough mastery of the various departments of pure mathematics, especially arithmetic, algebra, geometry, trigonometry, and conic sections. By the proper pursuit of these studies, the reasoning faculties of the mind are stimulated to action in the most direct and efficient, and yet simple manner conceivable.

This clearness of apprehension is not a little promoted by proper choice of language; and for this and other reasons, we must not fail to press upon the attention of the student the vast importance of the study of the languages, particularly the Latin and Greek. The character of the discipline which is derived from their well directed study, though differing from that resulting from scientific investigations, is nevertheless such as will be felt to be of eminent advantage throughout the individual's subsequent history. When we reflect that "language is the great instrument of human thought, the great means of communicating and receiving knowledge," and that in many instances the very clearness of our views depends in a measure upon our facility of giving them expression, we shall be led more carefully to consider the claims which the study of language has upon the attention of the student. Nor are we to forget that even a moderate knowledge of Latin and Greek greatly facilitates the acquisition of the different departments of natural and medical science, since it renders intelligible the technicalities of our profession, which would otherwise be arbitrary and without meaning. A good knowledge of the French and German languages would also be found of signal utility to the medical student and practitioner.

A writer in the British and Foreign Medical and Chirurgical Review remarks with much force and justice, that "the medical student, above all others, ought to be qualified to analyze with penetration, to compare with a clear perception of real analogy, to reason with logical precision, and to apply principles to practice with a ready apprehension," and for the development and training of these powers, he strongly recommends the well directed study of the various branches of natural science. These sentiments are also in accordance with the views of your Committee.

A judicious method of investigating the facts and principles of the natural sciences, will give the observing and comparative powers of the mind such an education as will enable the student, when he comes to examine the foundation studies of his professional curriculum, to take hold of them with such readiness and acuteness of apprehension as will give him an advantage of vast superiority over the student who attempts to grasp the same subjects without preparatory discipline. He will gain such broad and definite views as will cause his subsequent inquiries to be attended with much more philosophical discrimination, and consequent profit.

We repeat the idea, that it is of the first importance to the medical student, that his powers of observation and comparison be thoroughly developed. All his professional pursuits require a constant use of these faculties. Without their cultivation, with what advantage can he study the numberless and complex phases of disease, the therapeutical indications in each, and the just application of the various agents of the Materia Medica for curative effect?

Consider, for instance, the character of the discipline which would be gained by the proper pursuit of Botany, in all its departments, systematic, structural and physiological. In the first place, the simple collection of specimens for the purpose of analysis, induces the student to keep his eyes open on the objects about him, and thus begets in him a habit of vigilant observation, and of course counteracts the common and unfortunate tendency of passing through the world without seeing what is in it. Again: the study of that department of botany termed *organography*, or morphology, will admirably prepare him for the examination of the still more abstruse science of philosophical anatomy, a department of knowledge which is highly essential to a right understanding of the relative importance of the different parts of the human organism. Having examined structural botany, he is prepared to study the principles of classification and their application to what may be termed practical botany; and here, if the research is properly conducted, his powers of observation and discrimination are brought into active exercise. In these investigations the eye is educated, if we may so speak. It is taught to recognize the peculiarities of form, size, color, number and relative position of the various organs of the plant, and to compare these properties with those of other plants of the same order or genus, for the purpose of determining its genus or species. The faculties of comparison or discrimination are thus brought into active play, and if continued in use, will become so habituated to exercise as to be readily brought into action when the subsequent professional studies come under notice.

The same remarks may be made in reference to the influence on the mind of some other departments of natural science, particularly zoology, embracing, besides classification and external characteristics and habits of animals, their embryonic development, "organization, and the functions and inter-dependence on their component parts."

Besides the disciplinary influence of these studies, the information of a practical character which will be derived from them ought to be a sufficient inducement for their vigorous cultivation. There are very few, out of the whole number of physicians, who have even a general notion of these two sciences, especially the latter; and the knowledge they have acquired of them has been obtained, we venture to assert, in 9 cases out of 10, since the commencement of the active duties of their profession, and at a time too when they felt the stern necessity of more direct medical knowledge. But having constantly labored under a disadvantage from not possessing a knowledge of the elements of natural science, to say nothing of its extended details, they are willing or are compelled to make sacrifice of valuable time for the accomplishment of that which ought to have been the work of a part of their preliminary course.

We may notice for a moment the practical advantage to be derived from a knowledge of Botany. In the first place, it would enable the practitioner of medicine to recognize the indigenous medicinal plants of his own locality, and thus give him the means of furnishing himself several important agents of the different classes of the materia medica, as powerful as those imported, and much more sure of effect, since they can be used in their fresh state or before their virtues have been lost by exposure and keeping. It has been abundantly shown by American writers, "that our indigenous Materia Medica places within the reach of every practitioner, not only vegetable alteratives, diaphoretics, diuretics, expectorants, demulcents, tonics, anodynes, sedatives and astringents, but even emetics and cathartics, fully equal in value to those of any other country." The various plants included in these classes cannot, in many instances, be recognized by the popular names attached to them, since the same name is sometimes given to two plants of opposite botanical characters as well as therapeutic qualities; and hence the disadvantage and danger of relying on mere popular botanic nomenclature.

We need but refer, also, to the influence which a scientific practical botanist would have "in correcting the public mind and stopping the progress of the various species of '*vegetable*' quackery."

Thus, while the student is reaping wholesome discipline from the study of structural, physiological, and systematic botany, he is acquiring information which will be of direct application when he comes to enter upon the studies and the active duties of his chosen profession.

Similar observations may be noticed in reference to the practical applicability of the principles of zoology and comparative anatomy.

Natural philosophy should also enter into the studies of the primary course, since its principles are to be observed in estimating the character of certain physiological phenomena, and also in the practical operations of surgery.

A very desirable item in the education of the physician and surgeon, *is the education of the hand.* The ability with which many things are done, which come within the duties of the medical man, depends upon the accuracy, ease and expertness with which the hand can be used. In order to have the full advantages of manual education, a part of it should be obtained in the preliminary course. In order to this, we would recommend that due attention be given to the arts of drawing and modelling. Besides the valuable manual discipline which a practice of these arts would afford, it is easy to perceive, that in many exigencies of professional life, they could be brought into requisition with marked advantage.

Chemistry, although it is well taught in the medical schools, nevertheless fails of there being pursued in such a manner as to secure proficiency in the science. A writer in a recent number of one of the British Journals, expresses our views as to the position which chemistry should hold in the educational course. He says:—" We shall not, we trust, be accused of undervaluing chemistry as a branch of medical study, in saying that, in our opinion, the chemical course ought not to form a part of the purely professional system of education. For how stands the matter at present? The student either gives but little heed to it, picking up only as much knowledge as may be requisite to enable him to pass his examinations. Or if he feel interested in the pursuit, he devotes to it an amount of time and attention which can be illy spared from the more directly professional courses. In the former case, he loses the whole benefit of that mental discipline which the study of chemistry is peculiarly qualified to afford; in the latter, he loses valuable time, which he will find it difficult afterwards to replace. Morever, it is not from the course of chemistry as usually taught in our schools and colleges, that he gathers those refined applications of chemical doctrine which constitute the practical value of the study to the physician. The chemistry of physiology is, (or ought to be,) taught in the physiological course; that of pharmacy in the course on Materia Medica; that of pathology and therapeutics in the course on the Practice of Medicine. We would have even an increased amount of attention bestowed upon these topics; and the student would be more likely to come to them with a mind prepared to appreciate their value, if his attention had been given to chemistry as a science at that period of his educational course, at which it might be legitimately made a prominent object of pursuit, and at which the mastery of its principles would have the most beneficial influence on his intellectual character."

We, however, by no means propose wholly to exclude the teaching of philosophical chemistry from the medical college, but only advise that it be not wholly relied on as a means of acquiring chemical knowledge; and the more especially would urge this consideration while the lecture terms are, as at present, so short, and the whole medical course confined within the meagre limits of three short years.

The powers of observation would be still farther strengthened by a due attention to geology and mineralogy. The wide range of geological science, requiring, as it does, " an intimate acquaintance with the mineralogical history of the crust of the globe, and involving a series of distinct inquiries respecting the physical and chemical causes that are and have been producing the present and past changes," as well as " no small share of zoological and botanical knowledge,"—we say the wide range of this science affords in its study an ample opportunity for the application of the principles of chemistry and mineralogy, (which should be previously examined,) as well as botany, zoology and comparative anatomy, of course rendering these branches more familiar to the student, besides giving him discipline in the way of application of principles to a practical understanding of the science. We have not time to indicate the manner in which a knowledge of geology would be of direct service to the physician; but the well informed and reflecting mind will at once comprehend the bearings of the subject.

We would also urge upon the attention of the student, the pursuit of formal logic; for though his mathematical studies have been rightly conducted, and he has been instructed, during the acquisition of the other departments of his scientific course, relative to the reasoning process and the character of the fallacies with which he is likely to be embarrassed, he will still derive additional advantage from the examination of systematic treatises on the subject.

We cannot doubt, also, that a knowledge of mental philosophy should be insisted on as an essential prerequisite to the study of medicine. The intelligent physician need not be reminded of the intimate relations of mental phenomena to physical condition, and hence the value of a knowledge of the laws of mental manifestations. On this subject, the candidate for medical pupilage might be referred with confidence to such a work as Dr. ABERCROMBIE'S "Inquiries concerning the intellectual powers and the investigation of truth"

We would recommend, therefore, that before the student is received as a medical pupil, he be required to produce satisfactory evidence that, in addition to a thorough acquaintance with the elementary studies usually pursued in common schools, including a familiar knowledge of the principles and practice of English composition, he has also acquired such a knowledge of the Latin language as would result from a mastery of the grammar, and a critical reading of the Reader, the Georgics and Æneid of VIRGIL, the select orations of CICERO and LIVY, and such a knowledge of the Greek language as may be gained by a thorough acquaintance with the principles of its grammar, and their application to a careful reading of the Greek Reader and XENOPHON'S Anabasis. He should also be required to possess a good knowledge of Algebra, Geometry, Trigonometry, and Conic Sections; the various departments of Natural Philosophy, Philosophical Chemistry, Zoology, including Comparative Anatomy and Physiology, the arts of Drawing and Modelling, Mental Philosophy, Geology, Mineralogy, and Botany. * * * * * * * *

We are next to consider the *moral qualifications* which should be required of those who are eventually to assume the responsibilities of the physician. The life of the medical practitioner is one beset with peculiar temptations; and notwithstanding the serious associations connected with his professional duties, he is in frequent danger of attraction from the path of uprightness.

In these times, when charlatanry, in all its protean forms, stalks forth to compete with modest worth, it should be distinctly felt, that he who is subsequently to encounter the temptations incident to this unfortunate condition of things, should possess moral qualifications of something more than a negative character. However brilliant or profound the acquirements of the student, he should never be admitted as a pupil for medical instruction, unless he has undeniably established a reputation for sound moral worth; unless his integrity is of the most uncompromising character; unless his intelligence and morality combined are such, that he can be trusted amid the numerous temptations to unworthy ambition and sordid avarice. We would greatly prefer that he possess a puritanic rigidity of morals, than a mere passive innocence or negative goodness. His morality should be living, active, tangible. We would *prefer* that he possess the *true Christian character.* We would that his only written code of ethics need be the Golden Rule. But we cannot look for perfection: yet we *do* require that the student possess an undoubted reputation for unflinching integrity and high moral worth.

How are these considerations generally regarded in the admission of pupils into the office of the private practitioner? Is it not a well determined and lamentable fact, that in a majority of instances they are wholly disregarded? Not that we mean that in a majority of instances the persons admitted for instruction are morally unworthy, but that no thought is taken whether they are worthy or are not. It is not considered of sufficient importance to be made a matter of hesitation, and hence it cannot be concealed, that numerous persons are admitted to the professions who are morally unfit for its high duties and responsibilities. Who, that is acquainted with our medical colleges, does not know, that not a few in every class are distinguished, not for their scholarship, gentlemanly bearing and high moral worth, but for their ignorance, boorishness and vice? And how came they there? By the "advice and consent" of private practitioners, and whose certificates they have, attesting their faithful attention to medical science for so many years, and their good moral character. And if they graduate, as we are compelled with shame to admit that not a small number do, they graduate ignorant, boorish, and vicious. We have seen, at more than one college commencement, the profane, drunken libertine present himself before the President, by the side of a class-mate distinguished for his sound literary and scientific attainments and his elevated moral character, and there receive the highest honor known to our profession, and, thus "levelled up," go forth with his parchment as a fig leaf with which to cover the naked deformities of his character. These, we are no less willing than anxious to admit, are extreme cases, but they are indicative of the dangers which beset the true interests and honor of the profession, and the welfare of the community.

But it may be asked, how are we to ascertain the real character of the applicant? We answer, by duly investigating it; and we cannot be satisfied that it *is* what it should be, it is to be assumed that it is *not*. In many instances the *tout ensemble* of the individual's external manifestations, will enable us, at once, to determine upon his rejection. But where there is doubt, if we consider it worth the pains, we are to examine him as carefully as an ecclesiastical council would a candidate for holy orders. If he is found possessed of the requisite qcalifications, we need have but little fear as to his subsequent course; for it is to be presumed that, at the age when he is educationally prepared to commence his professional studies, his motives to action have acquired such permanency as to influence him, in a greater or less degree, throughout life.

On reviewing the considerations we have set forth, we are not unmindful of the fact that there are some who still question the propriety of our erection of what they are pleased to term a high standard. But we are unable, after a deliberate and impartial examination of the premises, to perceive what advantage is to result to the student, the profession, or the public, by the erection of a lower one. We cannot conscientiously lay the foundation narrower, lest the proportional superstructure be too frail and contracted to answer the purposes for which it was designed. Sooner than narrow down the foundations here indicated, we would make them deeper and broader; for it should be constantly borne in mind, that the measure of the student, when he enters our office, *cæteris paribus* is his proportional measure when he leaves the green room of the college.

It is preposterous to imagine that the responsibility of sending forth unqualified physicians rests wholly or even mainly upon the professors in our colleges. We send them rough, crooked, cross-grained and worm-eaten sticks, and then require them to return us sound, polished, and symmetrical pieces of furniture. We pour foul seed into their hoppers, and then absurdly demand that they return us fine flour and in good measure. We repeat the idea, that if the student possesses the literary, scientific, and moral qualifications we have indicated, it is prima facie evidence that he will not be inefficient in seeking those medical qualifications which will fit him for an intelligent and conscientious discharge of the duties of his profession. Upon private practitioners, therefore, mainly rests the responsibility of introducing to the profession, persons incapable of meeting its exigencies. We, *we* are the janitors at the temple gates of our Profession, and upon our vigilance and discrimination depend the character and usefulness of those who enter its honored portals.

